# Serpins in Tick Physiology and Tick-Host Interaction

**DOI:** 10.3389/fcimb.2022.892770

**Published:** 2022-05-19

**Authors:** Muhammad Nadeem Abbas, Adéla Chlastáková, Mohamed Amine Jmel, Evangelia Iliaki-Giannakoudaki, Jindřich Chmelař, Michail Kotsyfakis

**Affiliations:** ^1^ State Key Laboratory of Silkworm Genome Biology, Key Laboratory of Sericultural Biology and Genetic Breeding, Ministry of Agriculture, Southwest University, Chongqing, China; ^2^ Department of Medical Biology, Faculty of Science, University of South Bohemia in České Budějovice, České Budějovice, Czechia; ^3^ Laboratory of Genomics and Proteomics of Disease Vectors, Institute of Parasitology, Biology Centre of the Czech Academy of Sciences, České Budějovice, Czechia; ^4^ Laboratory of Molecular Biology of Ticks, Institute of Parasitology, Biology Centre of the Czech Academy of Sciences, České Budějovice, Czechia; ^5^ General Hospital of Heraklion Venizeleio-Pananeio, Heraklion, Greece

**Keywords:** tick saliva, serpins, immunomodulation, therapeutic effects, anti-tick vaccine, tick host interaction

## Abstract

Tick saliva has been extensively studied in the context of tick-host interactions because it is involved in host homeostasis modulation and microbial pathogen transmission to the host. Accumulated knowledge about the tick saliva composition at the molecular level has revealed that serine protease inhibitors play a key role in the tick-host interaction. Serpins are one highly expressed group of protease inhibitors in tick salivary glands, their expression can be induced during tick blood-feeding, and they have many biological functions at the tick-host interface. Indeed, tick serpins have an important role in inhibiting host hemostatic processes and in the modulation of the innate and adaptive immune responses of their vertebrate hosts. Tick serpins have also been studied as potential candidates for therapeutic use and vaccine development. In this review, we critically summarize the current state of knowledge about the biological role of tick serpins in shaping tick-host interactions with emphasis on the mechanisms by which they modulate host immunity. Their potential use in drug and vaccine development is also discussed.

## 1 Introduction

### 1.1 Tick-Host-Pathogen Triad

Ticks (order Ixodida) are ectoparasitic arthropods with a wide global distribution which serve as vectors of a broad spectrum of transmitted pathogens, including bacteria, viruses, and parasites. Ticks are medically considered the second most important vector of disease (Dantas-Torres et al., 2012). Ticks comprise two main families, soft ticks (Argasidae) and hard ticks (Ixodidae), with different lifestyles and life cycles, but both are obligate blood-feeders, entirely dependent on parasitic life. Their feeding strategies differ markedly; while hard ticks feed for several days until complete engorgement and repletion, soft ticks can complete their blood meal in less than one hour. Both groups of ticks alternately inject saliva and suck blood during this feeding process. Digestion takes place in the lumen of the midgut, where lysis of blood cells occurs, and subsequent digestion of proteins, including hemoglobin and other blood components, occurs intracellularly in the epithelial cells of the midgut. The process is driven by a cascade of intracellular endopeptidases and exopeptidases, in particular Cathepsins B, C, D, L and legumain, and leads to protein digestion down to single amino acids (Sojka et al., 2013).

Ticks penetrate the vertebrate skin with their saw-like hypostome, which serves to inject saliva and to draw blood but also opens the host skin to the external environment, leading to exposure to secondary infection. The resulting injury, transmitted pathogens, and superimposed infection trigger a host immune response. To avoid it, the tick releases its pharmacologically potent salivary constituents (Ribeiro and Mans, 2020) into the skin wound and alters all kinds of host immune responses. This action facilitates both tick feeding and pathogen transmission. The passage of transmitted pathogens within the tick tissues is usually described as pathogens entering the midgut from an infected host *via* the blood meal, then crossing the digestive epithelium and infiltrating the hemocoel, from where the pathogens can enter the salivary glands and infect the host while contained in tick saliva during the next feeding cycle (Šimo et al., 2017).

### 1.2 An Overview of Serpins

**Figure 1:**
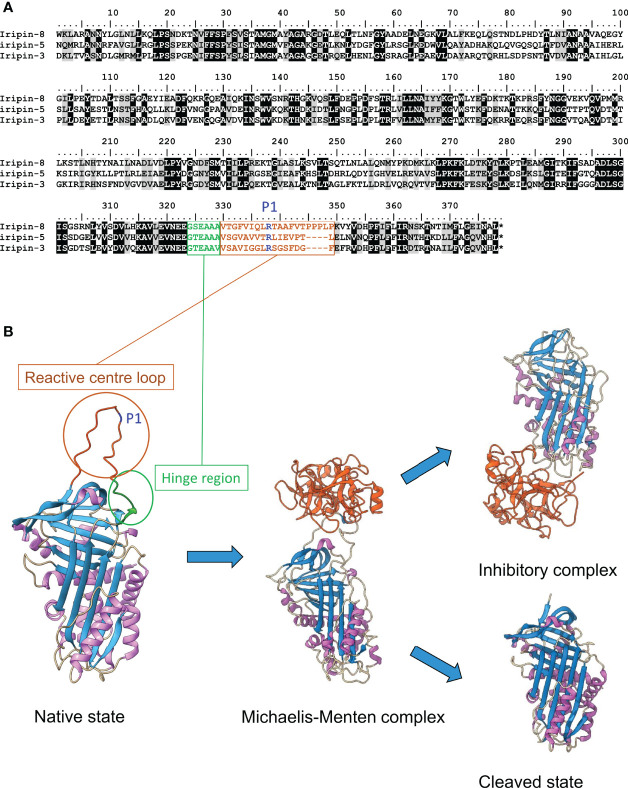
Structure of serpins and their mechanism of inhibition. **(A)** Sequence alignment of three characterized serpins from *I*. *ricinus*. RCL is highlighted in brown, P1 site in blue and hinge region in green. **(B)** Tertiary structures of four most common serpin conformation states. Native state is presented with highlighted RCL, P1 site and hinge region highlighted with the same colors as in the alignment. It forms non-covalent Michaelis-Menten complex with target protease, which can further end up as a covalent inhibitory complex or as cleaved inactive state. Used structures were downloaded from RCSB Protein Data Bank and prepared in ChimeraX (Pettersen et al., 2021). Asterisk in the alignment represents the stop codon.

Serpins form the richest group of serine (but they have been reported also as cysteine) protease inhibitors, consisting of 350-500 amino acid residues and ranging in molecular weight from 40 to 60 kDa. Recent and the most extensive phylogenetic study on serpins analyzed more than 18 000 unique protein sequences, extracted from public protein databases. Around 10 000 sequences differed by more than 25% in their amino acid sequence, showing enormous abundance of serpins among the organisms (Spence et al., 2021). Serpins are found mostly in eukaryotes, but they can also be detected in archaea, bacteria, and viruses, although in much smaller numbers than in eukaryotes, and many of them have also been functionally characterized. (Silverman et al., 2001; Gettins, 2002; Irving et al., 2002; Silverman et al., 2010; Spence et al., 2021). The number of serpin genes may vary in different animal species, and their distribution patterns in eukaryotes indicate that they appeared early in eukaryotic evolution (Logsdon et al., 1998). Inhibitory serpins usually play an important role in the regulation of physiological pathways controlled by serine proteases in vertebrates and invertebrates, including blood and hemolymph clotting, fibrinolysis, inflammation, complement activation, or regulation of the enzyme phenoloxidase in the Toll pathway in arthropods (Silverman et al., 2001; Rau et al., 2007; Gulley et al., 2013). Moreover, serpins are implicated in diverse biological processes in invertebrates, including immunoregulation, dorsal-ventral formation, development, and the regulation of apoptosis (Levashina Elena et al., 1999; Ligoxygakis et al., 2003; Pak et al., 2004; Kausar et al., 2017; Kausar et al., 2018). In plants, serpins are involved in the defense against insect pests and are studied for their application potential in agriculture (Alvarez-Alfageme et al., 2011; Clemente et al., 2019). In addition to their inhibitory role, serpins have been shown to modulate biological processes such as blood pressure or hormone transport in humans (Gettins, 2002; Zhou et al., 2006a; Whisstock et al., 2010). Interestingly, the hormone release mechanism is also dependent on the dynamics of serpin conformational changes (Zhou et al., 2008). Serine protease inhibitors are phylogenetically grouped by species rather than by their biological role in animals. Thus, rather than coevolution with serine proteases, the evolution of serine protease inhibitors appears to be driven by speciation in order to fulfill the species-specific biological roles (Krem and Di Cera, 2003). Despite relatively low sequence homology, all serpins have almost identical three-dimensional structure. This feature was explored in a recent phylogenetic study that suggested that convergent evolution has occurred several times in different taxa for serpins to acquire similar structure and function. The same study showed a high degree of conservation among intracellular serpins from both prokaryotes and eukaryotes, presumably with some key homeostatic function, whereas secreted serpins formed more species-specific branches (Spence et al., 2021). Thanks to protein crystallography, we have gained substantial insights into the molecular mechanism of serpin mode of action, which is termed suicidal because serpins form covalent complexes with the target protease(s) and are ultimately eliminated by a protein degradation mechanism (Whisstock et al., 2010; Huntington, 2011; Mahon and McKenna, 2018). As shown in **Figure 1**, serpins are composed of conserved β-sheets and α-helices and several coils that form a typical tertiary structure. Proper amino acid composition of specific region, called hinge region, allows the serpin to undergo necessary conformational changes that are crucial for their activity as protease inhibitors. A flexible, Reactive Center Loop (RCL) with P1 site functions as a bait for the target serine protease. It is exposed at the top of the serpin molecule and forms an intermediate Michaelis-Menten complex, which can further lead to the formation of covalent complex with the target protease. The final conformation of the serpin in the complex results from the insertion of the RCL into the β-sheet A to form one additional β-strand (Silverman et al., 2001; Gettins, 2002; Huntington, 2011). In case the inhibitory complex is not produced, cleaved serpin becomes inactive and active protease is released.

Despite the acronym serpin (Serine Protease Inhibitor) suggesting that serpins inhibit only serine proteases, it was experimentally shown that they could act as ‘cross-class’ inhibitors of proteases (Bao et al., 2018). For example, CrmA, a viral serine protease inhibitor, can inhibit caspase-1 protein (Komiyama et al., 1994) and SERPINB3 can inhibit cathepsins S, K, and L, which are papain-like cysteine proteases (Schick et al., 1998). In addition, miropin, a human pathogenic bacterial serpin, has been reported to inhibit a variety of both serine proteases, such as pancreatic and neutrophil elastases, cathepsin G, trypsin, plasmin or subtilisin and the cysteine proteases cathepsin L and papain (Ksiazek et al., 2015; Goulas et al., 2017; Sochaj-Gregorczyk et al., 2020). Such a wide inhibitory range could represent an adaptation strategy to the highly proteolytic environment of the subgingival plaque, which is constantly exposed to a number of host proteases in the inflammatory exudate. Under such environmental conditions, miropin is thought to play a key role as a virulence factor by protecting bacterial pathogens from the damaging activity of neutrophil serine proteases (Ksiazek et al., 2015). Miropin or CrmA are examples of the use of serpins by pathogens to invade and survive in the host. However, serpins are also used by blood-feeding arthropod ectoparasites to evade the host immune response and facilitate blood uptake.

## 2 Serine Protease Inhibitors in Ticks

Since the discovery of the serpin superfamily of serine protease inhibitors (Hunt and Dayhoff, 1980), many biological roles of serpins from different organisms have been discovered. Among other animals, many tick serpins have been identified using classical molecular methods, cDNA library screening or transcriptomic approaches (Ribeiro et al., 2012; Yu et al., 2013; Chmelař et al., 2016). In this review, we discuss tick serpins and their role in tick physiology and tick-host interactions in detail. We will focus on their anti-hemostatic, anti-inflammatory, anti-complement, and immunomodulatory functions in the host, and how these activities are important for pathogen transmission. Observed effects on the host are summarized in **Table 1** and inhibitory specificities, expressed by measured Ki values, are summarized in **Table 2**.

**Table 1:** Tick serpins examined in the current review article.

Serpin name	GenBank accession number	Tick species	Expression profile	Inhibited proteases	Biological processes affected by a serpin	Vaccination experiments	References
AamS6	ABS87358.1	*A. americanum*	Adult females, SG, saliva, MG, OVA	Trypsin, chymotrypsin, elastase, chymase, plasmin, papain	Platelet plug formation Coagulation cascade	–	Mulenga et al., 2007 Chalaire et al., 2011 Mulenga et al., 2013
AAS19	JAI08902.1	*A. americanum*	Adult females, SG, saliva, MG, OVA, SYN, MT	Trypsin, plasmin, fXa, fXIa, fXIIa, fIXa, thrombin, chymotrypsin, tryptase, papain	Platelet plug formation Coagulation cascade	Immunization of rabbits Reduced engorgement weight and impaired oviposion in adult female ticks	Porter et al., 2015 Kim et al., 2015 Kim et al., 2016 Radulović and Mulenga, 2017
AAS27	JAI08961.1	*A. americanum*	Eggs, larvae, nymphs, adults, SG, saliva, MG, CAR, OVA, SYN, MT	Trypsin, plasmin, chymotrypsin	Inflammation	–	Porter et al., 2015 Tirloni et al., 2019 Bakshi et al., 2019
AAS41	JAI08957.1	*A. americanum*	Eggs, larvae, nymphs, adults, SG, MG, CAR	Chymase, mast cell protease-1, chymotrypsin, papain	Inflammation	–	Porter et al., 2015 Bakshi et al., 2019 Kim et al., 2020
HLS-1	–	*H. longicornis*	Adult ticks, MG	–	Coagulation cascade	Immunization of rabbits Increased mortality rate in nymphs and adults	Sugino et al., 2003
HLS2	BAD11156.1	*H. longicornis*	Nymphs, adults, hemolymph	Thrombin	Coagulation cascade	Immunization of rabbits Prolonged feeding time and higher mortality rate in nymphs and adults, impaired oviposition	Imamura et al., 2005
HlSerpin-a	QFQ50847.1	*H. longicornis*	–	Cathepsin G, cathepsin B, fXa, papain	Inflammation Adaptive immunity	–	Wang et al., 2020
HlSerpin-b	QFQ50848.1	*H. longicornis*	–	Cathepsin G, fXa, papain	Inflammation Adaptive immunity	–	Wang et al., 2020
Ipis-1	BAP59746.1	*I. persulcatus*	Adult females, SG	–	Adaptive immunity	–	Toyomane et al., 2016
Iripin-3	JAA69032.1	*I. ricinus*	Nymphs, adult females, SG, saliva, OVA	Kallikrein, matriptase, thrombin, trypsin	Coagulation cascade Inflammation Adaptive immunity	–	Chlastáková et al., 2021
Iripin-5	JAA71155.1	*I. ricinus*	Nymphs, adult females, SG	Trypsin, elastase, proteinase-3	Inflammation Complement system	–	Kascakova et al., 2021
Iripin-8	ABI94058.1	*I. ricinus*	Nymphs, adult females, SG, saliva, MG	Thrombin, fVIIa, fIXa, fXa, fXIa, fXIIa, plasmin, activated protein C, kallikrein, trypsin	Coagulation cascade Complement system	–	Kotál et al., 2021
Iris	CAB55818.2	*I. ricinus*	Nymphs, adult females, SG, saliva	Elastase, tissue plasminogen activator, fXa, thrombin, trypsin	Platelet plug formation Coagulation cascade Fibrinolysis Inflammation Adaptive immunity	Immunization of rabbits Higher mortality and lower weight gain in nymphs, prolonged feeding period and higher mortality rate in adult females	Leboulle et al., 2002a Prevot et al., 2006 Prevot et al., 2007 Prevot et al., 2009
IRS-2	ABI94056.2	*I. ricinus*	Adult females, SG, MG, OVA	Chymotrypsin, cathepsin G, chymase, thrombin, trypsin, and other proteases	Platelet plug formation Inflammation Adaptive immunity	–	Chmelar et al., 2011 Páleníková et al., 2015 Pongprayoon et al., 2020 Fu et al., 2021
IxscS-1E1	AID54718.1	*I. scapularis*	SG, saliva, MG	Thrombin, trypsin, cathepsin G, fXa	Platelet plug formation Coagulation cascade	–	Mulenga et al., 2009 Ibelli et al., 2014
RAS-1	AAK61375.1	*R. appendiculutus*	Larvae, nymphs, adults, SG, MG	–	–	Immunization of cattle with a combination of RAS-1 and RAS-2 Decreased engorgement rate in nymphs, higher mortality in nymphs and adult females	Mulenga et al., 2003 Imamura et al., 2006
RAS-2	AAK61376.1	*R. appendiculutus*	Larvae, nymphs, adults, SG, MG	–	–		
RAS-3	AAK61377.1	*R. appendiculutus*	Male and female adults, SG, MG	–	–	Immunization of cattle with a combination of RAS-3, RAS-4, and RIM36 Higher mortality in female ticks	Mulenga et al., 2003 Imamura et al., 2008
RAS-4	AAK61378.1	*R. appendiculutus*	Male and female adults, SG, MG	–	–		
RHS-1	AFX65224.1	*R. haemaphysaloides*	SG, saliva	Chymotrypsin, thrombin	Coagulation cascade	–	Yu et al., 2013
RHS-2	AFX65225.1	*R. haemaphysaloides*	MG	Chymotrypsin	Adaptive immunity	–	Yu et al., 2013 Xu et al., 2019
RHS8	QHU78941.1	*R. haemaphysaloides*	Eggs, larvae, nymphs, adults, SG, OVA, fat bodies	–	Tick reproduction (vitellogenesis)	–	Xu et al., 2020
RmS-3	AHC98654.1	*R. microplus*	Nymphs, adult females, SG, saliva, MG, OVA	Chymotrypsin, cathepsin G, elastase, chymase, mast cell protease-1	Platelet plug formation Inflammation Adaptive immunity	–	Rodriguez-Valle et al., 2012 Rodriguez-Valle et al., 2015 Tirloni et al., 2014 Tirloni et al., 2014 Tirloni et al., 2016 Coutinho et al., 2020 Pongprayoon et al., 2021
RmS-6	AHC98657.1	*R. microplus*	Adult females, SG, saliva, MG, OVA	Trypsin, chymotrypsin, plasmin, fXa, fXIa	Inflammation	–	Tirloni et al., 2014 Tirloni et al., 2014 Rodriguez-Valle et al., 2015 Tirloni et al., 2016 Coutinho et al., 2020
RmS-15	AHC98666.1	*R. microplus*	Eggs, nymphs, adult females, SG, saliva, MG, OVA	Thrombin	Coagulation cascade	–	Tirloni et al., 2014 Tirloni et al., 2014 Rodriguez-Valle et al., 2015 Xu et al., 2016
RmS-17	AHC98668.1	*R. microplus*	Adult females, SG, saliva, MG, OVA	Trypsin, chymotrypsin, cathepsin G, plasmin, fXIa	Platelet plug formation Coagulation cascade Inflammation Adaptive immunity	–	Tirloni et al., 2014 Tirloni et al., 2014 Rodriguez-Valle et al., 2015 Tirloni et al., 2016 Coutinho et al., 2020

SG, salivary glands; MG, midgut; OVA, ovaries; SYN, synganglion; MT, Malpighian tubules; CAR, carcass; RIM36, Rhipicephalus immunodominant molecule 36 (a putative cement protein of R. appendiculatus ticks).

**Table 2:** Second-order rate constants of the interaction between tick serpins and serine proteases.

Serpin name	Tick species	Protease	Second-order rate constant (M^-1^ s^-1^)	References
AAS27	*A. americanum*	trypsin	6.46 ± 1.24 x 10^4^	Tirloni et al., 2019
AAS41	*A. americanum*	chymase	5.6 ± 0.37 x 10^3^	Kim et al., 2020
		α-chymotrypsin	1.6 ± 0.41 x 10^4^	
Iripin-3	*I. ricinus*	kallikrein	8.46 ± 0.51 x 10^4^	Chlastáková et al., 2021
		matriptase	5.93 ± 0.39 x 10^4^	
		trypsin	4.65 ± 0.32 x 10^4^	
		thrombin	1.37 ± 0.21 x 10^3^	
Iripin-8	*I. ricinus*	plasmin	2.25 ± 0.14 x 10^5^	Kotál et al., 2021
		trypsin	2.94 ± 0.35 x 10^4^	
		kallikrein	1.67 ± 0.11 x 10^4^	
		fXIa	1.63 ± 0.09 x 10^4^	
		thrombin	1.38 ± 0.1 x 10^4^	
		fXIIa	3.32 ± 0.41 x 10^3^	
		fXa	2.09 ± 0.12 x 10^3^	
		activated protein C	5.23 ± 0.35 x 10^2^	
		fVIIa + tissue factor	4.56 ± 0.35 x 10^2^	
Iris	*I. ricinus*	leukocyte elastase	4.7 ± 0.64 x 10^6^	Prevot et al., 2006
		pancreatic elastase	2.2 ± 0.15 x 10^5^	
		tissue plasminogen activator	2.9 ± 0.15 x 10^5^	
		fXa	1.7 ± 0.36 x 10^5^	
		thrombin	2.5 ± 0.42 x 10^4^	
		trypsin	1.5 ± 0.42 x 10^4^	
RmS-15	*R. microplus*	thrombin	9.3 ± 0.5 x 10^4^	Xu et al., 2016

All tick serpins with available data are presented.

### 2.1 Expression of Serpin Genes in Ticks

In ticks, serpins are usually expressed in different developmental stages and tissues but with some degree of stage and/or tissue specificity. For example, the serpin gene RHS8 has been shown to be expressed in all developmental stages, with mRNA levels being higher in *Rhipicephalus haemaphysaloides* larvae and nymphs (Yu et al., 2013; Xu et al., 2020). Similarly, serpins have been found to be transcribed in a number of tick tissues, suggesting a role either in tick physiology or in tick-host interactions. Such an interaction can occur either in the host or in the tick midgut. As an example, a study by Tirloni and co-workers analyzed the expression profiles of 18 serpins from *Rhipicephalus microplus* and found that 16 of them are transcribed in all tissues, but with quantitative differences for different serpins (Tirloni et al., 2014). Similarly, serpins from the Lone Star tick *Amblyomma americanum*, named Lospins, were also expressed in multiple tissues but with a tissue preference for individual serpins (Mulenga et al., 2007; Porter et al., 2015). The varying levels of expression across tissues suggest that serpins may have a broader biological role, i.e. serpins may be involved in development (present in the ovary) and in the regulation of blood digestion (present in the midgut). In addition, their expression and presence in salivary glands and/or saliva suggest that they play a role in tick feeding, possibly influencing host resistance mechanisms and facilitating pathogen transmission (Jmel et al., 2021). Therefore, in order to determine the role of individual serpins, we must not only investigate their capabilities in experimental models *in vitro* and/or *in vivo*, but we must also consider developmental stage and tissue specific expression, taking into consideration also the time during tick feeding that gene expression present peak(s). It is difficult to determine the concentration of tick salivary proteins in a host as the tick feeding site is a very complex and dynamic environment where the concentrations of both host and tick proteins constantly change (Mans, 2019). Therefore, we can only estimate roughly that the concentration of serpins can vary from nanomolar to micromolar range.

## 3 Serpins Modulate Tick Biological Processes Related to Disease Vector Physiology

**Figure 2:**
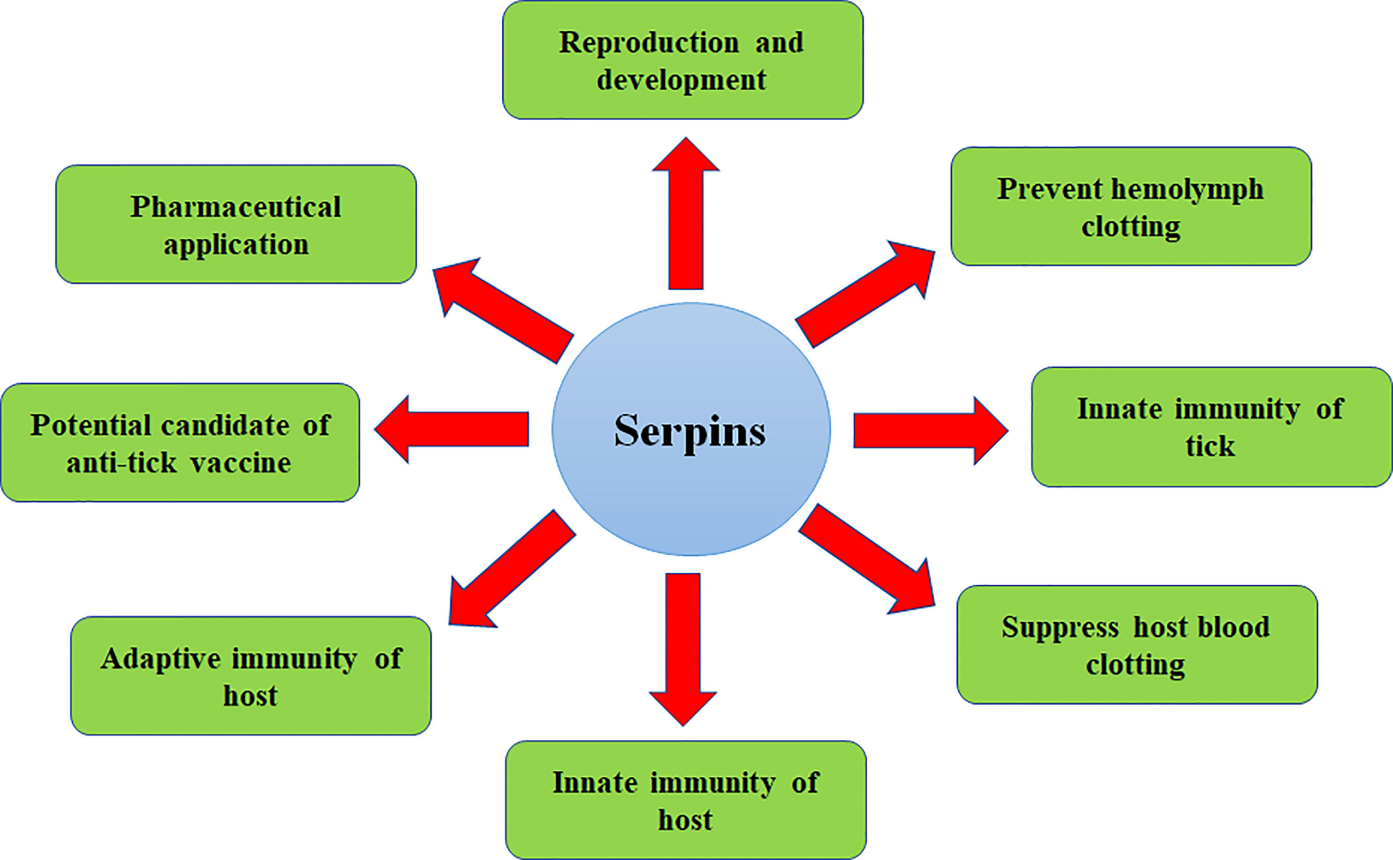
An overview of various biological processes that are regulated by serpins.

As discussed in the previous section, the pattern of serpin expression in different tick developmental stages and tissues may suggest a biological significance in tick physiology (**Figure 2** and **Table 1**). The first area in which serpins have a definite role is in the biology and physiology of ticks.

### 3.1 Serpins in Tick Hemolymph

In ticks and arthropods in general, hemolymph clotting is a key defense mechanism that reduces hemolymph loss and blocks entry into the wound, thereby preventing entry of microbial pathogens and tick infection/death. To date, several tick serpins have been identified as being involved in hemolymph clotting. The RAS-3 and RAS-4 serpins of the tick *Rhipicephalus appendiculatus* have been found to share some degree of similarity with the horseshoe crab hemolymph clotting factors LICI-1 and LICI-2, suggesting that they also have hemolymph clotting potential (Mulenga et al., 2003). Serpin HLS2, which is comparable to serpins from *R. appendiculatus*, was found to be produced only in the hemolymph, indicating that it likely controls processes in the hemolymph of this tick species (Mulenga et al., 2001; Imamura et al., 2005). Apart from exceptions, such as HLS2, it is not known, whether hemolymph serpins are produced by hemocytes or secreted there by other organs. Anyway, serpins are definitely produced in arthropod hemocytes, as they can be found by BLAST in hemocytomes not only from ticks (Kotsyfakis et al., 2015), but also from *Drosophila melanogaster* (BioProject database at NCBI, no. PRJEB33170).

In addition to coagulation, innate immunity processes are also present in the tick hemolymph. These processes contribute to the protection of ticks from pathogens and thus, are important factors in determining vector competence (Hajdušek et al., 2013). Several inhibitors of serine proteases have been reported to control the innate immune response in tick hemolymph, either by direct antimicrobial activity (Fogaça et al., 2006) or by a more complex role in arthropod immune response (Kopacek et al., 2012; Blisnick et al., 2017). Although serpins have not been experimentally proven to be involved in the tick immune response, their role in arthropod defense system was shown in *Anopheles stephensi*, in which serpin AsSRPN6 expression was induced by common microbiota bacterium *Enterobacter cloacae* and this correlated with inhibited development of *Plasmodium berghei* (Eappen et al., 2013). Thus, serpins can affect the composition of arthropod microbiota, which has direct implication in the defense against pathogens. Moreover, serpins are directly involved in the regulation of intracellular immune pathways, such as Toll pathway or myeloperoxidase production (Meekins et al., 2017). However, the main role of serpins in tick hemolymph appears to be in the regulation of proteolytic cascades, such as clot formation, rather than in the immune response *per se*.

### 3.2 Serpins as Regulators of Tick Reproduction

Another process related to tick physiology in which serpins play a role is oviposition. Serpins appear to be involved in tick reproduction alongside other key proteins such as vitellogenin or lipophorin (Tufail and Takeda, 2009). To date, many serpins have been identified as highly expressed in tick eggs and larvae (Andreotti et al., 2001; Sasaki et al., 2004). For example, the serpin RmS-3 is transcribed in the ovaries of *R. microplus* (Rodriguez-Valle et al., 2012; Rodriguez-Valle et al., 2015). *In vitro* feeding assays revealed that female ticks fed with anti-RmS-3 sheep serum had reduced egg weight and larval hatching rates, suggesting that RmS-3 is likely to be involved in tick reproduction and egg development (Rodriguez-Valle et al., 2012). The *R. microplus* serpins RmS-6, RmS-19, and RmS-20 might also play a role in tick embryogenesis or vitellogenesis (Rodriguez-Valle et al., 2015). The *R. haemaphysaloides* serpin RHS8 appears to stabilize vitellogenin by inhibiting serine protease activity since the knockdown of this serpin caused a significant reduction of vitellogenin protein levels, impaired oocyte maturation, and reduced fecundity (Xu et al., 2020). Similar evidence of serpin involvement in tick reproduction has been observed in *H. longicornis* (Zhou et al., 2006b) and *A. americanum* (Kim et al., 2016) when analyzing the effects of serpins on tick reproduction and development by vaccination experiments against tick serpins or RNA interference targeting serpin genes in these ticks.

### 3.3 Serpins as Regulators of Blood Fluidity and Digestion in Tick Midgut

Tick serpins might also be involved in the regulation of blood fluidity and digestion in tick midgut. This claim is supported by the fact that many serpins, some of which are known to possess anti-coagulant activity, have been found to be expressed in the midgut of feeding ticks (see **Table 1**). However, these functions have not yet been experimentally demonstrated. By employing a transcriptomic approach, Tirloni and his co-workers identified a total of 22 serpins in *R. microplus* (Tirloni et al., 2014; Rodriguez-Valle et al., 2015) with some of them (e.g. RmS-1, RmS-19, RmS-20, and RmS-21) being expressed in both the salivary glands and midgut, suggesting that certain *R. microplus* serpins might maintain blood in a fluid state at both the feeding site and in tick midgut and could regulate the process of blood meal digestion. Likewise, many serpins have been found to be expressed in both the salivary glands and midgut of feeding *A. americanum* ticks (Mulenga et al., 2007; Porter et al., 2015), and the same also applies to some *I. scapularis* serpins (Bakshi et al., 2018). HLS-1, the serpin of the tick *H. longicornis*, was revealed to be expressed only in the midgut of partially-fed ticks and had anti-coagulant activity in the aPTT (Activated Partial Thromboplastin Time) assay, which indicates that this particular serpin might be involved in maintaining blood fluidity in the midgut (Sugino et al., 2003).

## 4 The Importance of Tick Salivary Serpins in Tick-Host Interaction

Saliva is a complex mixture of various peptidic and non-peptidic components that are crucial for successful tick attachment. There are many reviews on the effects of tick saliva (Kotál et al., 2015; Šimo et al., 2017) and its individual components (Kazimírová and Štibrániová, 2013), including serine protease inhibitors (Blisnick et al., 2017; Chmelař et al., 2017). Serpins target hemostasis and the innate and adaptive branches of the host immune system. In the following sections, we will focus on the role of serpins in tick attachment success and how they modulate host immunity.

### 4.1 Tick Serpins Inhibit Host Hemostasis

#### 4.1.1 Host Hemostatic Response Against Tick Feeding

The first battle that a feeding tick must win is the battle against host hemostasis, a complex of host defense mechanisms that respond immediately to prevent blood loss from the physical injury caused by the tick mouthparts (once intruded into the host skin). Host hemostasis consists of vasoconstriction, plasma coagulation, and platelet aggregation. A number of cellular and biochemical processes take place in response to injury (LaPelusa and Dave, 2022). More specifically, after the resulting injury of the vascular epithelium, extrinsic clotting signaling is activated as epithelial cells begin to produce Tissue Factor (TF) to induce the clotting process. Tissue Factor interacts with pre-existing factor VIIa to form the TF-VIIa complex, which causes the cleavage of factor X. Factor XII activates a second intrinsic pathway in which high molecular weight kininogen and prekallikrein (PK) stimulate the cleavage of factors XI, IX, and the formation of the factor IXa-VIIIa complex, and the cascade ends with cleavage of factor X. Based on the above, it is clear that the activation cleavage of factor X to Xa is the target site of both coagulation pathways. The final product of both pathways is factor Xa, which binds to its cofactor Va and induces the prothrombinase complex. Finally, the factor Xa-Va complex converts factor II (prothrombin) to factor IIa (thrombin), which converts fibrinogen to fibrin and induces blood clotting (Jagadeeswaran et al., 2005; Kim et al., 2009).

Another process in hemostasis is platelet aggregation, which is an essential part of vertebrate defense against injury (Chmelar et al., 2011). Platelets are activated by contact with the extracellular matrix, which contains large amounts of adhesive macromolecules such as collagens and fibronectin (Jackson and Schoenwaelder, 2003; Furie and Furie, 2005; Watson et al., 2005). A number of surface protein interactions lead to the binding of the platelet GPVI receptor to collagen (Jandrot-Perrus et al., 2000). This causes integrins (e.g., α2β1) to switch to a high-affinity state, allowing them to mediate tight platelet adhesion to collagen while promoting the release of TXA2 and ADP, which are pro-inflammatory mediators (Jackson and Schoenwaelder, 2003; Furie and Furie, 2005; Watson et al., 2005). Vasoconstriction is the third hemostatic process mediated by smooth muscle cells and it is controlled by the vascular endothelium. Endothelial cells release molecules such as endothelin that control contractile properties of the blood vessels. Damaged blood vessels constrict to limit the amount of blood loss and the extent of bleeding. The presence of collagen exposed at the site of the damaged blood vessel promotes platelet adhesion. Salivary gland extract has been shown to impair vasoconstriction (Charkoudian, 2010; Pekáriková et al., 2015).

#### 4.1.2 Tick Serpins Target Host Blood Coagulation Factors

**Figure 3:**
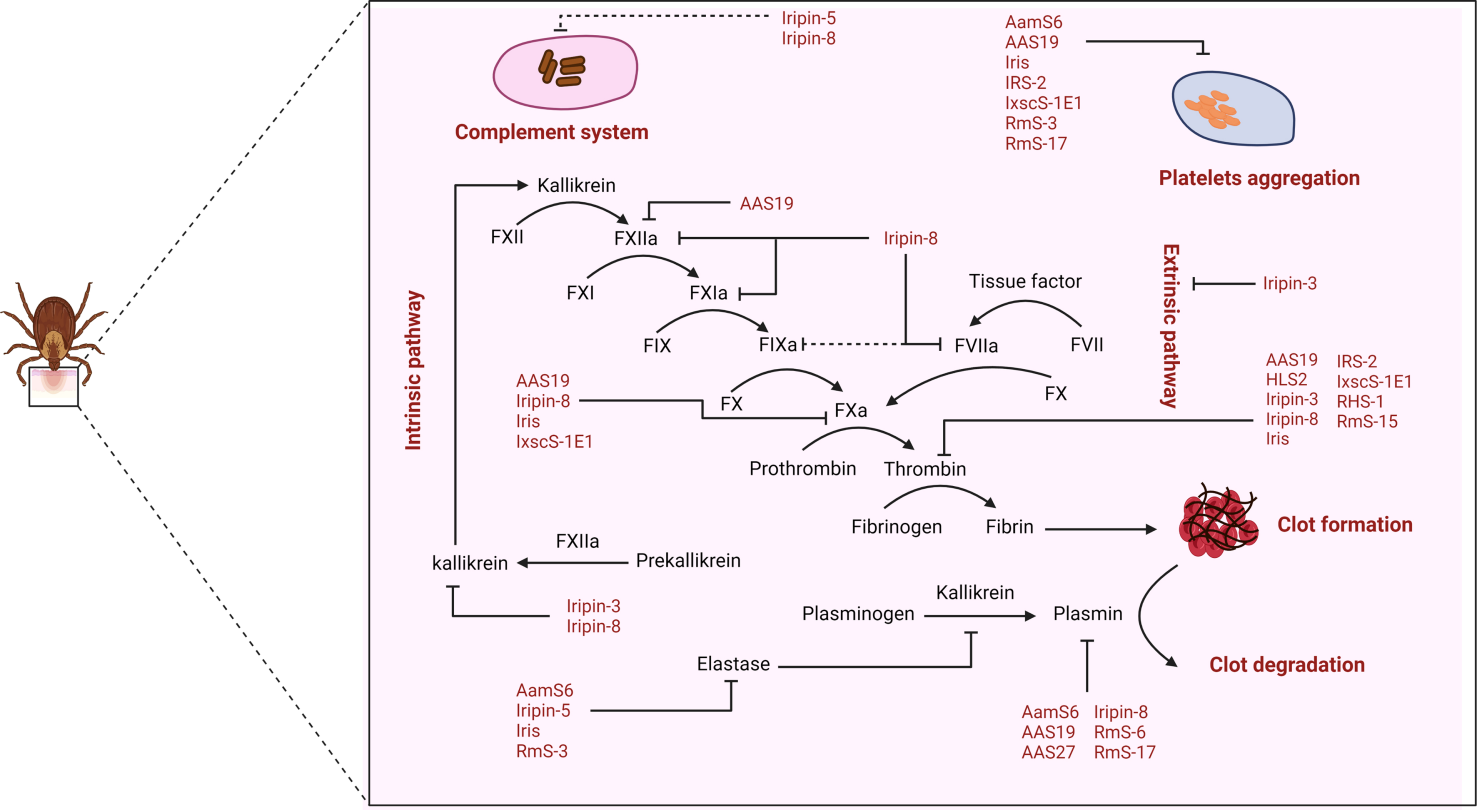
The role of tick salivary serpins in the regulation of host hemostasis and complement.

Ticks have developed a variety of molecules that they inject into the host *via* saliva to stop blood clotting (Chmelar et al., 2012). Since coagulation is a cascade of serine protease-dependent activations, inhibitors of serine proteases, including serpins, are the major regulatory factors involved in this process. In this section, we will discuss the molecular mechanisms that serpins use to inhibit blood clotting and to facilitate blood feeding (**Figure 3** and **Table 1**).

##### 4.1.2.1 Tick Serpins Interact With Host Thrombin

In vertebrates, thrombin is the main coagulation enzyme that catalyzes the conversion of fibrinogen to fibrin. Tick serpins are key regulators of this enzyme, as they control the balance between active and inactive thrombin. In ticks (but also in other hematophagous species), many thrombin inhibitors have evolved from different protein families, including serpins.

Of several serpins described and isolated from *R. microplus* (Rodriguez-Valle et al., 2015), only RmS-15 was found to substantially inhibit thrombin activity, as demonstrated by detailed enzymatic analysis (Xu et al., 2016). In addition, plasma clotting increased in the absence of serpin RmS-15, and higher titers of IgG antibodies to RmS-15 were detected in bovine serum after prolonged exposure to *R. microplus* challenge, suggesting its presence in tick saliva and its high immunogenicity (Rodriguez-Valle et al., 2015; Xu et al., 2016). The serpin RHS-1, which was identified from the closely related species *R. haemaphysaloides*, displayed strong expression in the salivary glands of fed ticks and inhibited chymotrypsin and thrombin activity *in vitro* (Yu et al., 2013). Consistent with its capacity to inhibit thrombin, RHS-1 prolonged plasma clotting time in the aPTT assay (Yu et al., 2013). These data suggest that RHS-1 may be involved in the inhibition of blood coagulation. Similarly, IxscS-1E1 is produced in both the salivary glands and midgut of *I. scapularis*, and its expression is increased after the first 24 h of tick feeding (Mulenga et al., 2009; Ibelli et al., 2014). This serpin formed stable complexes with thrombin and trypsin, inhibited platelet aggregation, and prolonged plasma clotting time, as demonstrated by *in vitro* experiments (Ibelli et al., 2014). The serpin Iripin-8 from *I. ricinus* also inhibited thrombin and other proteases of the coagulation cascade and it has been shown to be a potent inhibitor of the intrinsic and common pathways of the coagulation cascade, as evidenced by aPTT and TT (Thrombin Time) assays (Kotál et al., 2021). Other, rather weak inhibitors of thrombin from the same tick species are Iris (Prevot et al., 2006), IRS-2 (Chmelar et al., 2011), and Iripin-3 (Chlastáková et al., 2021). However, additional data on these serpins suggest a role other than anticoagulation.

##### 4.1.2.2 Tick Serpins Regulate Host Blood Coagulation via Inhibition of FX(A) and Other Blood Clotting Factors

Activated factor X (FXa) is a central enzyme of coagulation that stands at the intersection of both coagulation activation pathways and is responsible for the activation of thrombin (Borensztajn et al., 2008). To date, several FX(a) inhibitors, including serpins, have been described in various tick species. In 2002, the first serpin named Iris (*Ixodes ricinus* immunosuppressor) was isolated from the tick *I. ricinus* (Leboulle et al., 2002b). Besides other immunomodulatory effects and the aforementioned inhibition of thrombin, Iris inhibited factor FXa in a dose-dependent manner and with higher specificity than thrombin (Prevot et al., 2006). Serpin Iripin-8 also inhibits factor FXa and other proteases of the coagulation cascade, including factors fVIIa, fIXa, fXIa, fXIIa, APC (activated protein C), kallikrein, and thrombin, demonstrating that it is an inhibitor of coagulation by targeting many different host enzymes at the same time (Kotál et al., 2021). Iripin-3 has also been shown to block coagulation, but only the extrinsic pathway. Thus, Iripin-3 was the first tick serpin to inhibit this type of coagulation activation (Chlastáková et al., 2021). The serpin AAS19, which was originally identified by RNA sequencing of *A. americanum* and is expressed in the salivary glands and midgut during tick feeding (Porter et al., 2015), was found to be able to inhibit a wide range of proteases of the coagulation cascade, such as FXa and FXIa. Reduced activity of the same serpin was also reported against FXIIa, FIXa and thrombin (Kim et al., 2015).

##### 4.1.2.3 Inhibition of Fibrinolysis by Tick Serpins

Fibrinolysis is a highly regulated enzymatic process that prevents the unnecessary accumulation of intravascular fibrin and enables the removal of thrombi (Chapin and Hajjar, 2015). The cleavage of insoluble fibrin polymers into soluble fibrin degradation products is mediated by plasmin that is generated from the zymogen plasminogen by either tissue-type plasminogen activator (tPA) or urokinase-type plasminogen activator (uPA) (Schaller and Gerber, 2011; Chapin and Hajjar, 2015). Plasmin, tPA, and uPA are serine proteases whose enzymatic activity is commonly regulated by serpins, such as plasminogen activator inhibitor-1, plasminogen activator inhibitor-2, and α2-antiplasmin (Schaller and Gerber, 2011; Chapin and Hajjar, 2015). Some tick serpins, e.g. *A. americanum* serpins AAS19 and AAS27 (Kim et al., 2015; Tirloni et al., 2019). *I. ricinus* serpin Iripin-8 (Kotál et al., 2021), and *R. microplus* serpin RmS-17 (Tirloni et al., 2016), reduced the proteolytic activity of plasmin *in vitro*; however, their effect on fibrinolysis has not been tested. The only tick serpin that has been shown to inhibit fibrinolysis thus far is Iris derived from the tick *I. ricinus* (Prevot et al., 2006). The anti-fibrinolytic effect of Iris is probably mediated though its ability to inhibit tPA since Iris devoid of any anti-protease activity due to a mutated RCL did not significantly affect fibrinolysis time (Prevot et al., 2006). Even though tick serpins can reduce the enzymatic activity of plasmin and tPA, the inhibition of fibrin clot dissolution makes no sense in the context of blood feeding since it is in tick’s best interest to maintain host blood in a fluid state both at the feeding site and in tick midgut. However, beyond fibrinolysis, plasmin is also involved in the inflammatory response (Syrovets et al., 2012), as described later in this review in the section 4.2.2., dedicated to the effects of tick serpins on host inflammation. Unlike the aforementioned inhibition of fibrin clot dissolution, attenuation of inflammation by targeting plasmin might be beneficial for feeding ticks.

##### 4.1.2.4 Tick Serpins and Their Interaction With Glycosaminoglycans

The inhibitory activity of some serpins involved in the regulation of blood coagulation and fibrinolysis can be altered by their interaction with glycosaminoglycans (GAGs), such as heparin or heparan sulfate (Gettins, 2002; Huntington, 2003; Rau et al., 2007). GAGs can influence the anti-proteolytic activity of serpins in two ways. First, they can simultaneously bind both the serpin and the protease, bringing them together in an appropriate orientation for the productive interaction of the serpin’s RCL with the protease active site (Gettins, 2002). Second, GAGs binding to the serpin can lead to the alteration of the serpin conformation to one in which the serpin is more reactive toward the target protease (Gettins, 2002). The *A. americanum* serpin AAS19 has four predicted GAG-binding sites on its surface, suggesting it could be responsive to GAGs (Kim et al., 2015). Indeed, binding of heparan sulfate/heparin to AAS19 caused pronounced changes in the inhibitory profile of the serpin in that AAS19 inhibitory activity was significantly increased against thrombin and FIXa and was considerably reduced against FXa and FXIIa. Overall, AAS19 interaction with GAGs enhanced the capacity of this serpin to suppress the coagulation cascade (Radulović and Mulenga, 2017). It is likely that this observation is just an example of how glycosaminoglycans are involved in the regulation of tick serpins activity and more examples would be found if we focused in that direction.

#### 4.1.3 Platelet Aggregation and Tick Serpins

Platelet aggregation is necessary for the formation of hemostatic plugs. It is a complex and dynamic multistep adhesion process involving various receptors and adhesion molecules, especially integrins (Jackson, 2007; Li et al., 2012). Importantly, platelet aggregation can be triggered by certain serine proteases, such as cathepsin G and thrombin. Cathepsin G, which is released by activated neutrophils, can induce platelet aggregation through the activation of protease-activated receptor-4 (PAR-4) (Sambrano et al., 2000), and blood clotting factor thrombin can trigger platelet aggregation by activating PAR-1 and PAR-4 (Lisman et al., 2005). Tick serpins that were shown to reduce the enzymatic activity of cathepsin G and/or thrombin, such as *A. americanum* serpin AAS19 (Kim et al., 2015), *I. ricinus* serpin IRS-2 (Chmelar et al., 2011), *I. scapularis* serpin IxscS-1E1 (Ibelli et al., 2014), or *R. microplus* serpins RmS-3 and RmS-17 (Tirloni et al., 2016) inhibited *in vitro* platelet aggregation triggered by these two serine proteases (see **Table 1**). This suggests that tick serpins can suppress primary hemostasis through their capacity to inhibit serine proteases involved in the activation of platelet aggregation. However, the inhibitory effect of some tick serpins on platelet plug formation might be independent of their anti-proteolytic activity. For example, the RCL mutants of the serpin Iris from the tick *I. ricinus* lost their anticoagulant activity but still managed to inhibit platelet adhesion (Prevot et al., 2006). As discussed in this particular study, serpins may interact *via* exosites with other proteins such as von Willebrand factor and integrins to block platelet adhesion on endothelial cells (Prevot et al., 2006; Berber et al., 2014). Overall, tick serpins appear to have an important role in inhibiting platelet adhesion, thus blocking the specific host response to tick feeding, but other salivary protein families are also known to mediate the same effect.

### 4.2 Tick Serpins Regulate Host Innate Immunity

**Figure 4:**
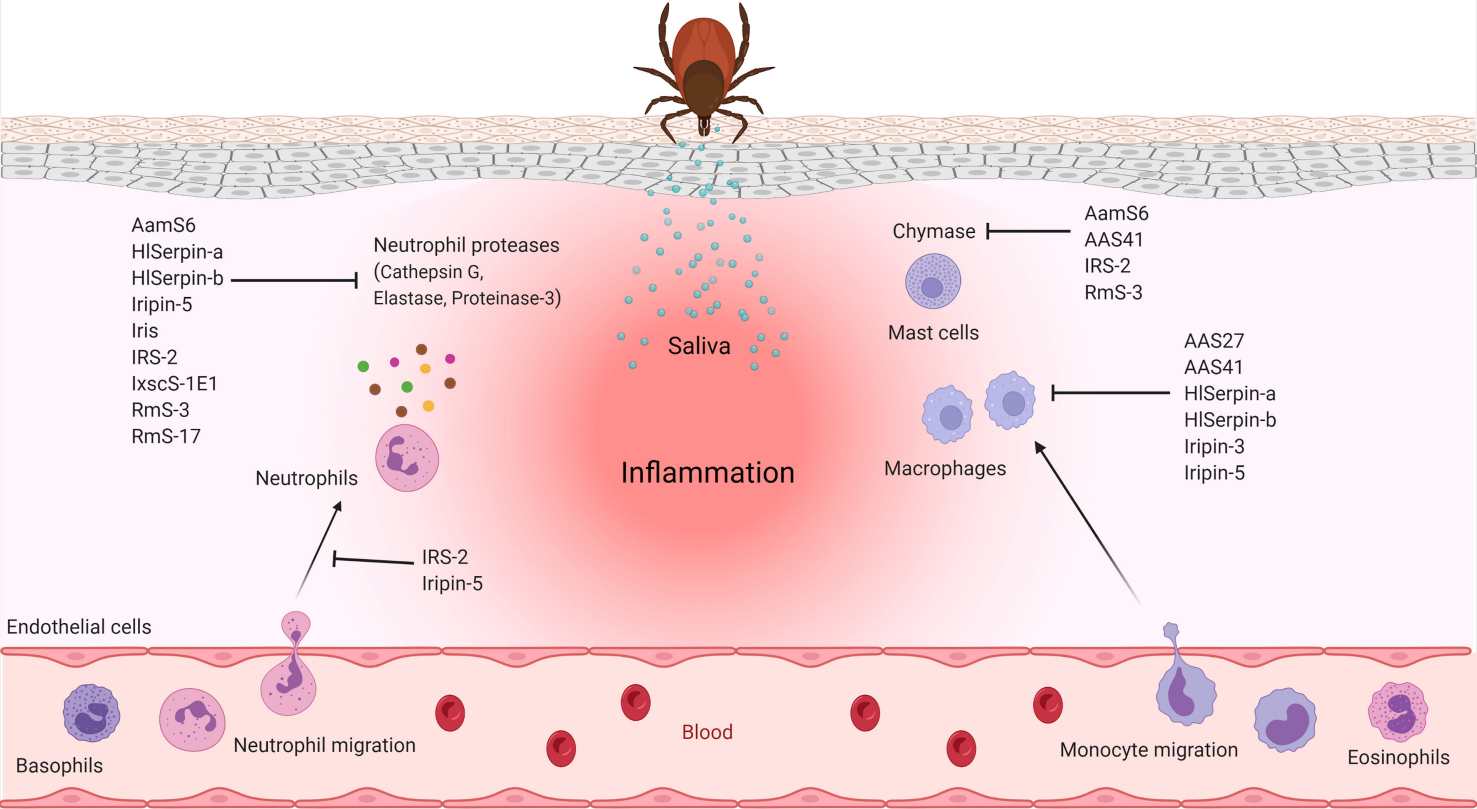
Anti-inflammatory activities of tick salivary serpins.

**Figure 5:**
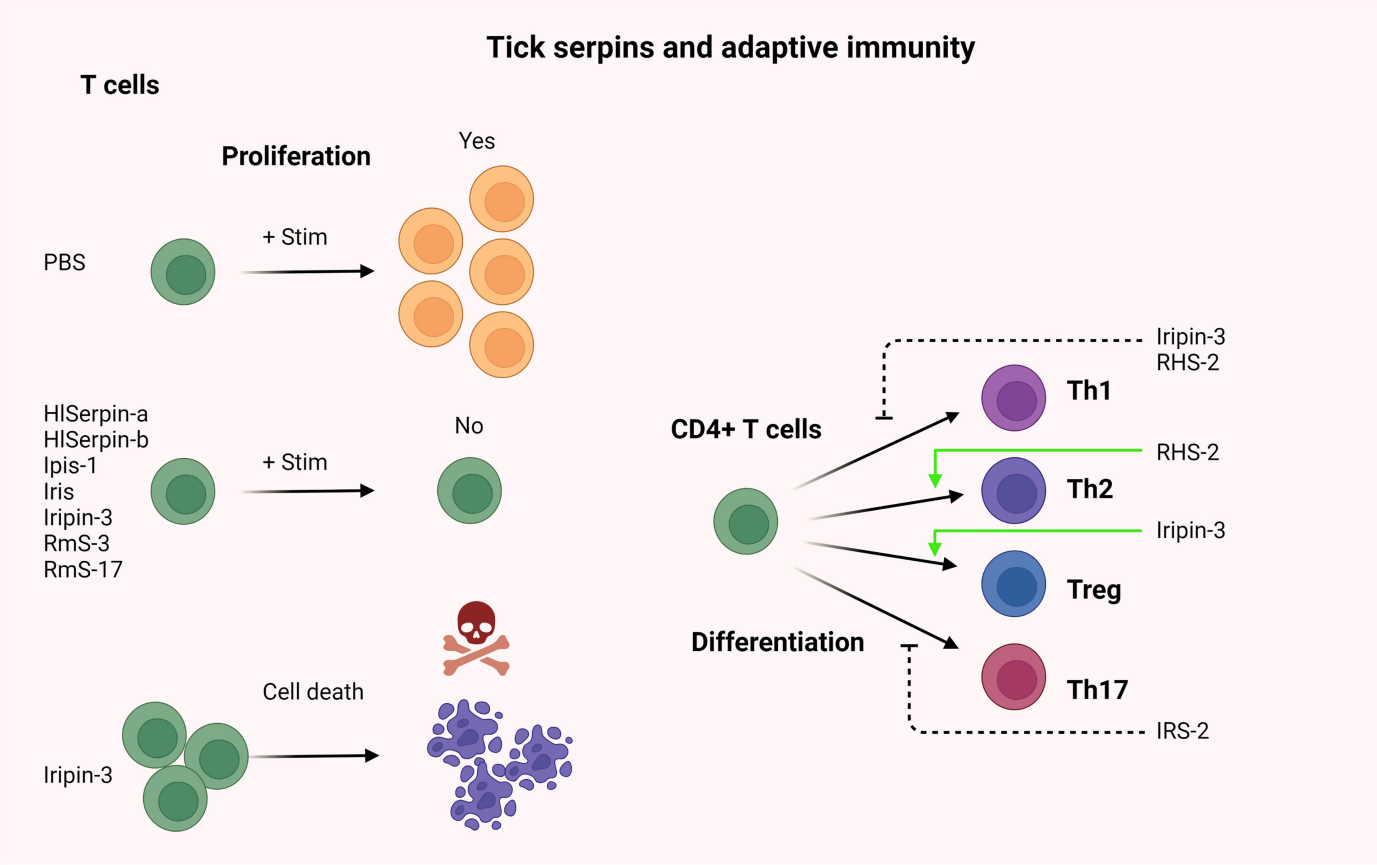
The role of tick serpins in the modulation of vertebrates host adaptive immune system.

Injury caused by a tick hypostome, together with concomitant and/or transmitted infections, induces a host immune response, which begins with the activation of pattern recognition receptors (PRRs) by pathogen- or danger-associated molecular patterns (PAMPs or DAMPs). Activated resident cells begin to produce cytokines and chemokines that recruit from the bloodstream to the site of injury/infection various innate immune cells, such as neutrophils and monocytes. Complement activation further amplifies the local inflammatory response. The feeding period, which extends to several days in Ixodidae, provides sufficient time for the development of adaptive immunity, which includes both humoral and cellular branches. To prevent rejection by the host, ticks use a mixture of pharmacologically active molecules at the site of injury to manipulate all types of host immune responses. Many excellent and thorough review articles have been published describing both the immune response against tick attachment and the effects of tick saliva or of individual salivary compounds on the host immune system (Hovius et al., 2008; Francischetti et al., 2009; Kazimírová and Štibrániová, 2013; Kotál et al., 2015; Chmelař et al., 2016; Chmelař et al., 2017; Kazimírová et al., 2017; Šimo et al., 2017; Chmelař et al., 2019; Wen et al., 2019; Aounallah et al., 2020; Martins et al., 2020; Fogaça et al., 2021; Jmel et al., 2021; Narasimhan et al., 2021; Wikel, 2021; Wang and Cull, 2022). In the following section, we discuss how tick salivary serpins contribute to the evasion of immunity-mediated host defense mechanisms – both innate (**Figure 4**) and adaptive (**Figure 5**).

#### 4.2.1 Tick Serpins and Host Complement

The vertebrate complement system enhances the ability of phagocytic cells to remove microbial pathogens and damaged cells by opsonization, by promoting inflammation and by directly attacking cell membrane components of pathogens (Kimura et al., 2009; Cagliani et al., 2016). Tick saliva and its protein components possess anti-complement activity, which has been reported in several publications (Schroeder et al., 2009; Miller et al., 2011; Cong et al., 2013; Wikel, 2013). So far, a number of anti-complement proteins have been discovered in the saliva of several tick species. A well-characterized complement inhibitor that binds the C5 component and thereby inhibits its activation by C5 convertase has been isolated from the soft tick *Ornithodoros moubata* (Fredslund et al., 2008). It inhibited complement-mediated hemolytic activity as well as the development of pathological features in a rodent model of myasthenia gravis (Hepburn et al., 2007). Other tick complement inhibitors, such as Isac, Irac-1, and -2, and Salp20, belong to the ISAC/IRAC family of proteins and inhibit the alternative complement pathway by binding and displacing properdin, thereby inhibiting C3 convertase production (Valenzuela et al., 2000; Schroeder et al., 2007; Tyson et al., 2007).

Recently (and for the first time in ticks), anti-complement activities have been described for two *I. ricinus* serpins, namely Iripin-5 and Iripin-8 (Kascakova et al., 2021; Kotál et al., 2021), and their anti-complement activity was comparable to that of vertebrates serpins (Bos et al., 2002; Kascakova et al., 2021; Kotál et al., 2021). Iripin-5 has a dose-dependent inhibitory activity against complement system, as evidenced by a decrease in erythrocyte lysis when incubated with increasing concentrations of Iripin-5 (Kascakova et al., 2021). Iripin-8 serpin exhibited a similar effect, but approximately 10-fold weaker anti-complement activity when compared to Iripin-5 (Kotál et al., 2021). In summary, these findings suggest that tick serpins may also be involved in complement inhibition at the tick attachment site. However, further studies would be required to unravel the molecular mechanism by which these serpins regulate the complement cascade.

#### 4.2.2 Tick Serpins and Host Inflammation

The role of serpins in the regulation of inflammation is well known because the most abundant serpin in human serum is alpha-1-antitrypsin, which is a major protective factor against the damaging effects of neutrophil elastase (Mangan et al., 2008; Yaron et al., 2021). Other human serpins, such as antichymotrypsin, also have an anti-inflammatory function. Not surprisingly, many serpins from tick saliva exhibit anti-inflammatory effects in both *in vitro* and *in vivo* experiments. These activities are thought to result from their inhibitory specificity towards important pro-inflammatory proteases such as neutrophil elastase, cathepsin G, plasmin or chymase.

Plasmin is a key protease in hemostasis, particularly in fibrinolysis, but it is also involved in the development of the inflammatory response by playing a major role in producing proinflammatory cytokines, in regulating monocyte and dendritic cell chemotaxis, and in attracting neutrophils to the site of inflammation (Syrovets et al., 2012). Several tick serpins inhibited plasmin, but the association between this inhibition and the observed anti-inflammatory phenotype has not been directly demonstrated. Antiplasmin specificity has been observed in serpins from *A. americanum* - AamS6, AAS19, AAS27 (Chalaire et al., 2011; Syrovets et al., 2012; Mulenga et al., 2013; Kim et al., 2015; Bakshi et al., 2019). In a recent study, the serpin AAS27 was found to have a peak of expression at 24 h after tick attachment and formed SDS-stable irreversible complexes with trypsin and plasmin and blocked both formalin- and compound 48/80-induced inflammation in rats. Thus, AAS27 appears to be an anti-inflammatory protein, but the causal link to plasmin inhibition is not yet demonstrated (Tirloni et al., 2019). The most potent plasmin inhibitor among tick serpins so far is *I. ricinus* serpin Iripin-8 (Kotál et al., 2021), which however showed no immunomodulatory or anti-inflammatory effect in several assays.

Neutrophil elastase is one of the four neutrophil serine proteases with a key role in killing bacteria and in activating inflammatory mediators. Its inhibition should be beneficial to ticks. *I. ricinus* serpin Iris inhibited several elastase-like proteases, including leukocyte and pancreatic elastase, and also exhibited anti-inflammatory effects, but these were explained by exosite activity (Leboulle et al., 2002a; Prevot et al., 2006; Prevot et al., 2009). Another elastase inhibitor from *I. ricinus* is Iripin-5, which affects neutrophil migration, decreases nitric oxide production by macrophages, and modifies complement function, thus exhibiting potent anti-inflammatory activity (Kascakova et al., 2021). Anti-elastase activity was described for the other two tick serpins, namely AamS6 and RmS-3 (Chalaire et al., 2011; Syrovets et al., 2012; Mulenga et al., 2013; Pongprayoon et al., 2021).

Under normal physiological functions, mast cells are known to regulate vasodilation, vascular homeostasis, innate and adaptive immune responses, and angiogenesis (Krystel-Whittemore et al., 2016). Large granules in the cytoplasm of mast cells store inflammatory mediators, including histamine, heparin, a variety of cytokines, chondroitin sulfate, and neutral proteases, like chymase and tryptase (Moon et al., 2014). Cathepsin G and chymase, which are produced after mast cell activation, regulate the acute inflammatory response, particularly during the cross-talk of IL-2 between neutrophils and platelets (Zarbock et al., 2007). These proteases are often targeted by tick serpins, indicating their importance in host defense against tick feeding. Mast cell chymase affects inflammation at multiple levels, including cleavage of proinflammatory cytokines/chemokines and activation of protease-activated receptor 2, degradation of endothelial cell contacts, activation of extracellular matrix-degrading enzymes, and recruitment of eosinophils/neutrophils (Pejler et al., 2010). Serpin RmS-3 from *R. microplus* tick saliva inhibited rMCP-1, the major chymase produced by rat connective tissue-type mast cells in the peritoneum (Coutinho et al., 2020). It has also been shown that serpin RmS-3 reduces vascular permeability stimulated by compound 48/80, which can cause degranulation of plantar-type mast cells, thermal hyperalgesia, tissue edema, and neutrophil infiltration (Chatterjea et al., 2012). Thus, RmS-3 may be a key component in modulating the early steps of inflammatory reactions by blocking the chymase which is generated during mast cell activation (Coutinho et al., 2020). Chymase also appears to be crucial for the degradation of tick anticoagulants, so its inhibition should help the tick to maintain blood fluidity (Fu et al., 2021). A recent study showed that the serpin IRS-2 of *I. ricinus* can inactivate almost all connective tissue chymases from a range of animals, including human, hamster, rat, dog, and opossum, as well as mucosal mast cell proteases, rat blood vessel chymases, and also neutrophil proteases. However, this serpin fails to inactivate mast cell tryptases and the basophil-specific protease mMCP-8 (Fu et al., 2021). The first study of the tick serpin IRS-2 disclosed the protein as having a preferential specificity for chymase and cathepsin G and as having a significant anti-inflammatory effect *in vivo* by reducing swelling and neutrophil migration into inflamed tissues, while a later study showed that IRS-2 reduced spirochete *Borrelia burgdorferi*-induced IL-6 production in splenic dendritic cells (Chmelar et al., 2011; Páleníková et al., 2015). Moreover, IRS-2 impaired the development of proinflammatory Th17 cells by reducing STAT-3 phosphorylation (Páleníková et al., 2015). Overall, by inhibiting mast cell chymase, IRS-2 can affect host inflammatory response against tick feeding.

#### 4.2.3 Tick Serpins and Host Cytokines

Cytokines play a central role in the communication between host immune cells, in their differentiation and maturation, and in the overall control of the immune response. Tick serpins altered the production of various cytokines in many experiments, modulating the immune response, mostly from a pro-inflammatory to an anti-inflammatory direction.

*Haemaphysalis longicornis* serpins HlSerpin-a and HlSerpin-b can suppress the expression of pro-inflammatory cytokines such as TNF-α, interleukin (IL)-6, and IL-1β from lipopolysaccharide (LPS)-induced mouse bone marrow-derived macrophages or mouse bone marrow-derived dendritic cells (BMDCs) (Wang et al., 2020). Furthermore, this study demonstrated that cathepsins B and G are required for sufficient LPS stimulated activation of mouse macrophages (Wang et al., 2020). This suggests that tick serpins may use their protease inhibitory activities to suppress the activation of host immune cells.

In addition, two serpins from *A. americanum* (AAS27 and AAS41) were shown to regulate evasion of host immune response by altering host cytokine secretion (Bakshi et al., 2019). Based on the results of this study, it seems that *A. americanum* saliva proteins can be divided into two groups, those with LPS-like activity causing the expression of pro-inflammatory (PI) markers by macrophages and those that suppress the expression of pro-inflammatory markers in activated macrophages. The PI group included the insulin-like growth factor binding-related proteins (AamIGFBP-P6S, AamIGFBP-P1, and AamIGFBP-P6L). These PI recombinant proteins could stimulate PBMC (peripheral blood mononuclear cell) derived macrophages and mouse RAW 267.4 macrophage lineage *in vitro*. Following this activation, PI co-stimulatory markers, such as CD40, CD80, and CD86, and cytokines (e.g. TNF-α, IL-1, and IL-6) were produced by these macrophages. In contrast, *A. americanum* tick salivary anti-inflammatory (AI) serpins, including AAS27 and AAS41, did not affect cytokine expression or PI markers production by macrophages. However, AI serpins could enhance the expression of AI cytokines (TGFβ and IL-10) in macrophages pre-activated by LPS or PI recombinant proteins. In addition, the injection of PI-tick salivary proteins (individually or as a cocktail) into mice induced paw edema *in vivo*, resulting in increased levels of CD40, CD80, CD86, IL-1, TNF-α, IL-6, and chemokines (CCL2, CXCL1, CCL3, CCL5, and CCL11). In comparison, the AI serpins AAS27 and AAS41 (cocktail and individually) suppressed the activation of host immune cells. Overall, PI proteins activated host immune cells towards the production of pro-inflammatory cytokines, whereas AI serpins inhibited such production, implying that ticks may use a combination of PI and AI proteins to evade host immune defenses (Bakshi et al., 2019).

### 4.3 Tick Serpins Regulate Host Adaptive Immunity

Vertebrates are the only group with “Darwinian” type of adaptive immunity (Muller et al., 2018). This type of immunity is based on a large number of pre-formed clones with a wide range of specificities, which is able to further increase its accuracy in response to antigens. In anti-tick immunity, the adaptive branch plays a role, especially later during the feeding course in the case of primary exposure to ticks, but also earlier in the case of repeated tick infestation on the same host. During this process, a plethora of cytokines is released, each of which is responsible for steering towards distinct types of immune responses. Pro-inflammatory response mediated by Th1 cells have a crucial role in the defense against pathogen infection and is deleterious also for tick feeding (Raphael et al., 2015; Hirahara and Nakayama, 2016; Duan et al., 2019; Ng et al., 2021).

Several tick serpins were shown to modulate adaptive immunity (**Figure 5**), affecting mostly CD4+ T cell proliferation, survival, and differentiation to T cell subpopulations, but also the production of many cytokines. Iripin-3 from *I. ricinus* disrupted the survival and proliferation of CD4+ T cells; moreover, it suppressed the differentiation of T helper type into pro-inflammatory Th1 cells and promoted the differentiation into T regulatory cells (Chlastáková et al., 2021). Finally, the same study showed that Iripin-3 reduced the generation of the pro-inflammatory cytokine interleukin-6 by bone marrow-derived macrophages activated with LPS. Thus, Iripin-3 appears to be another pluripotent salivary serpin with immunomodulatory and anti-hemostatic properties that can facilitate tick feeding by suppressing host anti-tick immune reaction (Chlastáková et al., 2021). Some of these observations are similar to those with Iris, which also suppressed CD4+ T cell proliferation and the production of pro-inflammatory cytokines IFN-γ, IL-6, TNF-α, and IL-8 (Leboulle et al., 2002a). Dendritic cells play crucial role in the adaptive immunity as they can affect, which direction the immune response will proceed. Ticks can alter the biology of dendritic cells as described previously (Sa-Nunes and Oliveira, 2021). *R. haemaphysaloides* derived serpin RHS-2 blocked the differentiation of bone marrow-derived cells into dendritic cells while promoting the differentiation of these cells into macrophages. RHS-2 also inhibited dendritic cell maturation and the expression of CD80, CD86, and MHC-II. Moreover, this serpin suppressed the differentiation of Th1 cells, as evidenced by decreased production of the cytokines IL-2, IFN-γ, and TNF-α (Xu et al., 2019). The serpin Ipis-1 has been shown to be expressed in the salivary glands of unfed and feeding *Ixodes persulcatus* ticks and was reported to be associated with immunomodulatory effects on the acquired immune responses (Toyomane et al., 2016). More specifically, Ipis-1 inhibited the proliferation of bovine peripheral blood mononuclear cells (PBMCs) and IFN-γ production (Toyomane et al., 2016). However, the precise molecular mechanism behind the aforementioned Ipis-1 inhibitory activities is not known (Toyomane et al., 2016).

The immune cells that have been activated acquire additional biological roles such as cytokine production, proliferation, and chemotaxis (Moro-García et al., 2018; Zhang et al., 2020). A recent study analyzed the ability of *R. microplus* serpins RmS-3, RmS-6, and RmS-17 to reduce the metabolic activity of splenocytes and the production of the cytokine IFN-γ (Coutinho et al., 2020). This study showed that in the presence of 1 µM RmS-3, concanavalin A (ConA)-stimulated spleen cells displayed a partial decrease in their metabolic activity, whereas RmS-6 had no impact on the metabolic activity of these cells (Coutinho et al., 2020). RmS-17 serpin also lowered the metabolic activity of ConA-stimulated spleen cells in a dose-dependent manner, with a substantial effect at 300 nM and 1 µM concentrations (Coutinho et al., 2020). IFN-γ production in ConA-stimulated splenocytes treated with *R. microplus* serpins followed similar patterns. RmS-3 used at 1 μM concentration partially inhibited IFN-γ production, RmS-6 did not modify it, and RmS-17 strongly inhibited IFN-γ production at both 300 nM and 1 µM concentrations (Coutinho et al., 2020). The authors of the same study also investigated the effects of these three serpins on the proliferation of T lymphocytes. They showed that naïve T lymphocytes did not proliferate when incubated with medium or in the presence of RmS-3, RmS-6, and RmS-17 serpins alone. Under suboptimal activation conditions, T lymphocytes exhibited weak proliferation, which was partially inhibited in the presence of RmS-3, not affected by RmS-6, and completely inhibited by RmS-17 (Coutinho et al., 2020). However, under optimal activation conditions, RmS-3 and RmS-6 had no significant effect on the robust proliferation of T lymphocytes, and RmS-17 managed to inhibit T cell proliferation only partially (Coutinho et al., 2020). Overall, it seems that some tick serpins can suppress T cell proliferation and IFN-γ production to produce optimal conditions for tick feeding on vertebrate hosts. However, more research is needed to better understand this phenomenon and its molecular mechanism.

## 5 Tick Serpins Are Promising Molecules for Therapeutics Development

The presence of swollen joints indicates that there is an increase in the amount of fluid in the tissues around the joints. People who suffer from different types of arthritis, infections, and injuries may have swollen joints. A recent study has shown that tick serpins can also be used as a substance to treat these ailments. However, full-length serpins, which contain about 400 amino acids, have a number of disadvantages for use in drug development (Wang et al., 2020). The reactive center loop of serpins is the main inhibitory region that directly binds to serine proteases (Huntington et al., 2000; Whisstock et al., 2010), but without a conserved tertiary structure, the inhibitory potential of RCL should be lost. In a rather surprising study, Wang and co-workers synthesized a peptide corresponding to the RCL of HlSerpin-a from *H. longicornis* (Wang et al., 2020). The authors suggested that the minimal active region (i.e. RCL) of this tick serpin has similar inhibitory activity and immunosuppressive properties as the whole serpin. In a mouse arthritis model, the RCL peptide derived from HlSerpin-a substantially impaired cytokine production from immune cells and alleviated joint swelling and tissue inflammation. This preliminary observation surprisingly suggests that the RCL of a functional tick serpin could be used as a drug, because of its non-immunogenic nature due to small size and easy synthesis (Wang et al., 2020).

## 6 Tick Serpins as Epitopes for Anti-Tick Vaccine Development

Ticks are effective vectors of a variety of viral and bacterial diseases in vertebrates. Therefore, ticks are studied extensively all over the world in order to develop management strategies to control them or to immunize vertebrate hosts against ticks. Some pesticides (e.g., acaricides) are routinely used to control tick populations (Nwanade et al., 2020). However, pesticides drastic impacts on non-target species, the evolution of resistant tick populations, and the resulting environment hazard are the major concerns against the use of pesticides (Nwanade et al., 2020). Researchers around the globe are attempting to develop environmentally friendly and sustainable strategies to control ticks. For example, the development of a vector-specific vaccine may immunize (and protect) the vertebrate hosts but also may have a detrimental influence on tick population growth in the areas where (immunized) host activity is localized. Many laboratories work on the potential development of vaccines that would use tick-derived epitopes. These vaccines should be effective in tick control while simultaneously reducing the transmission of viral or bacterial pathogens (**Table 1**).

Many molecules have been tested as targets for the development of such vaccines. Serpins that are found in a wide range of animals, including ticks, appear to be promising targets. Imamura and colleagues injected a mixture of two recombinant serpins (RAS-1 and -2) from *R. appendiculatus* into cattle for the first time. Nymphs and adult ticks that fed on the cattle immunized by these two serpins had higher mortality rates, and the egg-laying capacity of the female ticks was also reduced when compared to the control group. However, the feeding time of the ticks was approximately identical on both the vaccinated and unvaccinated hosts (Imamura et al., 2006). Another salivary serpin, Iris from *I. ricinus*, was also examined as a potential anti-tick vaccine target. Prévot et al. administered recombinant Iris protein into mice and rabbits, but only rabbits developed anti-tick immunity as evidenced by higher mortality and lower weight gain in nymphs and by a prolonged feeding period and a higher mortality rate in adult females (Prevot et al., 2007). Most of the functionally characterized tick serpins, such as RmS-3, AAS41, and others, have been suggested as prospective vaccine candidates (Kim et al., 2020; Pongprayoon et al., 2021). However, the majority of these serpins have not been evaluated in vaccination experiments (see **Table 1**). Therefore, further investigations are required to advance the vaccine development process.

Even though it has been established that the administration of some serpins can improve the immunity of the host against ticks, the way to get considerably higher levels of protection is to produce vaccines based on multiple members of the serpin family. Individual differences in the expression of different members of the serpin family may make it possible to target a larger number of ticks. Another possibility is to prepare anti-tick cocktail vaccine by combining members of different protein families. For example, Imamura et al. immunized cattle with a combination of *R. appendiculatus* serpins RAS-3 and RAS-4 and a putative cement protein RIM36 (Imamura et al., 2008). The administration of this coctail vaccine led to an increased mortality of female ticks feeding on immunized cattle (Imamura et al., 2008). Moreover, immunization of a host with serpins conserved in many different tick species (such as *I. ricinus* serpin Iripin-8, *A. americanum* serpin AAS19, *R. microplus* serpin RmS-15, and *R. haemaphysaloides* serpin RHS8) might be a more efficient strategy than relying on the serpins present only in a small number of closely related tick species since the conservation of these serpins suggests they might play an important role in tick biology. It was suggested previously that tick salivary proteins undergo some kind of antigenic variation in order to escape from the recognition by host adaptive immune system and that there is a redundancy in salivary proteins functions (Chmelar et al., 2016). Therefore, in order to prepare an effective vaccine, conserved epitopes or the cocktail with multiple antigens should be used. An interesting opportunity came up from the lesson we learned about mRNA vaccines during the Covid19 pandemic. Recently a research group employed a mixture of mRNAs coding for tick salivary proteins as an anti-tick vaccine, and they observed very promising effects against the transmission of *B. burgdorferi* (Matias et al., 2021; Sajid et al., 2021). It will be interesting to observe the development of new types of anti-tick vaccines in this direction.

## 7 Future Perspectives

The interactions between arthropod parasites such as ticks and their hosts have always been of interest. Ticks developed strategies to evade host defensive response in order to successfully complete a blood meal. Ticks serve as a reservoir of pathogens that are transmitted to the host during blood feeding. In recent years, advances in molecular techniques have made it possible to investigate the factors which mediate this interaction, providing a much-needed impetus to unlock previously unattainable insights into this phenomenon. A better molecular understanding of this phenomenon will help in the development of methods to identify a subset of antigens that could be used as potential vaccine targets. Many of the serpins identified are involved in various biological processes in ticks. Serpins also play a role in the maintenance of blood fluidity by inhibiting thrombin, FXa, and other factors. They are also involved in controlling the innate and adaptive immune responses of the host. Several serpins have been shown to be effective candidates for enhancing host anti-tick immunity.

Serpins display multiple functions in various *in vitro* and *in vivo* experiments. Their functional characterization usually requires recombinant proteins. Fortunately, functional recombinant serpins are usually relatively easy to produce in large quantities in bacterial expression system. This system, however, does not take into account possible post-translational changes. The mechanisms behind the observed effects are usually not known for tick serpins and this is the direction we should focus on in future studies. Their inhibitory mechanism can be altered by point mutation of P1 site, thus the indispensability of inhibitory function of serpins can be tested. According to published data, serpin RCL alone can display interesting activity (Wang et al., 2020) and application potential. Since the function of serpins is mostly dependent on structural changes, structural analyses could be employed in mechanistic studies as well. Finally, serpins represent great material for protein engineering to gain novel functions, as shown both for inhibitory and non-inhibitory serpins (Chan et al., 2014; Polderdijk et al., 2017).

Serpins definitely have application potential in drug development. Inflammation is a symptom of a variety of diseases, and currently available therapies are limited. Researchers are looking for natural compounds with potent anti-inflammatory activities and novel chemical structures. Ticks and other blood feeding arthropods can be considered as a rich source of proteins with unique biological activities against vertebrate homeostasis. Tick serpins appear to be useful for treatment of inflammatory diseases (Wang et al., 2020). Although the data are rather preliminary to support drug development based on tick serpins, further research can help to identify other medically relevant serpins and to translate the laboratory studies into preclinical and clinical trials. Finally, there is some evidence to suggest serpins as potential candidates for vaccine development against ticks at least as a part of the vaccine cocktail.

## Author Contributions

MA, JC and MK designed the structure of the article. All authors performed the literature search and wrote parts of the manuscript/assembled the data. MA, AC and JC extracted the data and prepared the tables. MA, JC and AC created and edited the figures. MK made critical revisions and proofread the manuscript. All authors read and approved the final manuscript.

## Funding

This work was supported by Grant Agency of the Czech Republic (grant 19-14704Y to JC and 22-18424M to AC). MK received funding from the Grant Agency of the Czech Republic (grant 19-382 07247S) and ERD Funds, project CePaVip OPVVV (No. 384 CZ.02.1.01/0.0/0.0/16_019/0000759). The funders had no role in the design, data collection and analysis, decision to publish, or preparation of the manuscript.

## Conflict of Interest

The authors declare that the research was conducted in the absence of any commercial or financial relationships that could be construed as a potential conflict of interest.

## Publisher’s Note

All claims expressed in this article are solely those of the authors and do not necessarily represent those of their affiliated organizations, or those of the publisher, the editors and the reviewers. Any product that may be evaluated in this article, or claim that may be made by its manufacturer, is not guaranteed or endorsed by the publisher.
